# Ensuring the continuity of essential health services during mpox response in the African region

**DOI:** 10.4102/jphia.v16i1.1227

**Published:** 2025-03-31

**Authors:** Miriam Nanyunja, Viviane Fossouo, Ebenezer Obi Daniel, Joseph C. Okeibunor, Samuel Boland, Hilary K. Njenge, Brice W. Bicaba, Otim P.C. Ramadan, Solomon F. Woldetsadik, Dick D. Chamla, Fiona Braka, Abdou S. Gueye

**Affiliations:** 1 World Health Organization, Regional Office for Africa, Emergency Preparedness and Response Hub, Nairobi, Kenya; 2 World Health Organization, Regional Office for Africa, Emergency Preparedness and Response Cluster, Brazzaville, Democratic Republic of the Congo; 3 Africa Centre for Disease Control Headquarters, Addis Ababa, Ethiopia

## Introduction

The mpox epidemic across the World Health Organization (WHO) African Regions highlights the critical need for concerted efforts to ensure the continuity of essential health services (CEHS) during health emergencies.^1^ These services include immunisation, reproductive, maternal, neonatal and child health (RMNCH) care, the management of non-communicable diseases (NCDs), and the treatment of chronic infectious diseases such as human immunodeficiency virus (HIV) and tuberculosis.^2^ Disruptions of these services during emergencies have significant long-term effects on health outcomes, increasing the vulnerability of populations and compounding existing challenges. Public health stakeholders in the WHO African Region must assess the maintenance of CEHS during emergencies and initiate corrective measures early to strengthen health systems’ resilience.

## Lessons from the past: Ebola and COVID-19

The 2014–2016 West Africa Ebola Virus Disease (EVD) outbreak exposed significant weaknesses in the health systems of affected countries.^3^ The crisis underscored the importance of robust health system pillars, community engagement and strong institutional leadership. Because of the absence of these elements, many countries experienced disruptions in CEHS, which is reflected in poor performance on relevant indicators. For example, human resources for health, a key driver of CEHS, were severely impacted, with high healthcare worker mortalities in Sierra Leone, Guinea and Liberia. Additional issues included poor health information management, inadequate availability of medicines, weak governance and insufficient health financing, all of which slowed the response to the outbreak.^4^

The COVID-19 pandemic presented even greater challenges, particularly for countries with weak health systems. Efforts to curb the disease significantly disrupted CEHS, negatively affecting economic stability and biopsychosocial well-being. While the African region reported fewer cases and deaths than other regions, the pandemic’s impact was still substantial. Preparedness levels before the pandemic were crucial in determining the ability to maintain CEHS. However, by 2018, many countries were unprepared for outbreaks with pandemic potential, with only 32.6% of Member States in the WHO African Region demonstrating the capacity to maintain CEHS and just 6% of these countries were classified as fragile, conflict-affected and vulnerable.^5^

A global assessment of COVID-19 preparedness and response plans across 106 countries revealed that most prioritised disease surveillance (99%), laboratory testing (96%) and COVID-19 case management (97%). However, only 47% considered CEHS planning, 34% addressed sub-national continuity of health service delivery, 24% ensured the quality of care, and 7% had a monitoring and evaluation plan for CEHS.^6^ Fear of contracting COVID-19 deterred non-COVID-19 patients, particularly those with comorbidities such as hypertension, diabetes and tuberculosis, from seeking care, and worsening clinical outcomes also impacted CEHS during the pandemic.^7,8^ Health worker redeployment to pandemic response efforts further reduced the availability of CEHS for these patients, potentially increasing non-COVID-19 attributable deaths.^9,10^ These findings highlight the need for prioritising primary healthcare as part of emergency preparedness and response strategies.

## Maintaining continuity of essential health services during the mpox response

In the context of the ongoing mpox epidemic, a desk review was conducted to assess preparedness and response plans in 14 Member States. The review focused on five key areas: inclusion of a CEHS pillar, CEHS-related objectives, activities to ensure CEHS, budgetary allocations and indicators for monitoring CEHS. Only four countries (28.5%) integrated CEHS considerations into their plans, with significant budget allocations and monitoring indicators gaps. Building on lessons from past outbreaks for maintaining CEHS during the mpox response requires strategic and integrated approaches to minimise disruptions while effectively managing the outbreak.^11^

Key strategies include comprehensive planning, which entails developing integrated strategies for maintaining CEHS, tailored to varying mpox prevalence across countries; integrating mpox response activities with routine healthcare services, including RMNCH, immunisation programmes, and care for NCD patients; increasing the number of healthcare workers in infectious disease clinics by providing focused training and implementing standard referral systems and mobilise alternative workforces, including retirees and students from learning institutions, in high-prevalence areas.^12,13^ Community engagement and communication demand attention. This will address fear among non-mpox patients by conducting public health campaigns that communicate safety measures at health facilities; engage community leaders and organisations to disseminate preventive guidelines and maintain trust in health services and equip community health workers to provide home care for non-critical mpox cases, reducing the burden on healthcare facilities.^14,15,16,17,18,19^

Strengthening surveillance and data systems is critical for implementing real-time data collection and monitoring of mpox cases and CEHS utilisation to ensure efficient resource allocation. Leveraging digital health technologies will facilitate case tracking, resource management and workforce optimisation. Adopting data-driven approaches will enhance the resilience of health systems during the mpox response and future emergencies.^20,21^

## Conclusion

The mpox epidemic underscores the importance of learning from past outbreaks to prevent disruptions in CEHS. Despite progress in essential medicine supply chains, digital health technologies and health system resilience, significant gaps remain. Governments and public health partners in the WHO African region must integrate CEHS strategies into emergency response plans, supported by adequate funding and infrastructure investments. Strengthening community engagement and health system infrastructure is vital to protect routine health services during emergencies. Implementing these measures will mitigate the long-term impacts of infectious disease outbreaks and improve health outcomes for African populations.
